# Receptor ligand-triggered resistance to alectinib and its circumvention by Hsp90 inhibition in EML4-ALK lung cancer cells

**DOI:** 10.18632/oncotarget.2055

**Published:** 2014-06-03

**Authors:** Azusa Tanimoto, Tadaaki Yamada, Shigeki Nanjo, Shinji Takeuchi, Hiromichi Ebi, Kenji Kita, Kunio Matsumoto, Seiji Yano

**Affiliations:** ^1^ Divisions of Medical Oncology, Cancer Research Institute, Kanazawa University, Kanazawa, Japan; ^2^ Tumor Dynamics and Regulation, Cancer Research Institute, Kanazawa University, Kanazawa, Japan

**Keywords:** Hsp90 inhibitor, EML4-ALK, drug resistance, receptor ligands, New generation ALK inhibitor.

## Abstract

Alectinib is a new generation ALK inhibitor with activity against the gatekeeper L1196M mutation that showed remarkable activity in a phase I/II study with *echinoderm microtubule associated protein-like 4* (*EML4*) - *anaplastic lymphoma kinase* (*ALK*) non-small cell lung cancer (NSCLC) patients. However, alectinib resistance may eventually develop. Here, we found that EGFR ligands and HGF, a ligand of the MET receptor, activate EGFR and MET, respectively, as alternative pathways, and thereby induce resistance to alectinib. Additionally, the heat shock protein 90 (Hsp90) inhibitor suppressed protein expression of ALK, MET, EGFR, and AKT, and thereby induced apoptosis in EML4-ALK NSCLC cells, even in the presence of EGFR ligands or HGF. These results suggest that Hsp90 inhibitors may overcome ligand-triggered resistance to new generation ALK inhibitors and may result in more successful treatment of NSCLC patients with EML4-ALK.

## INTRODUCTION

Non-small cell lung carcinoma (NSCLC) can be classified into distinct molecular subsets based on specific genomic alterations that drive tumorigenesis [1]. *ALK* rearrangement, most commonly *EML4-ALK*, is detected in approximately 3–7% of unselected NSCLCs [2, 3]. *EML4-ALK* NSCLC is more frequently observed in patients with adenocarcinoma than with other diseases, in young adults than in older patients, and in non-smokers or light smokers (<15 packs/year) than in heavier smokers [4]. Crizotinib, a multiple tyrosine kinase inhibitor (TKI) of ALK, MET, and ROS1, is the only agent that has been approved for *ALK*-rearranged NSCLC. It shows dramatic clinical efficacy, with a response rate of about 60–80% and a progression free survival (PFS) of approximately 9–10 months in *ALK*-rearranged NSCLC patients [5]. However, almost all patients who strongly responded to crizotinib acquired resistance to these agents after varying periods of time [6].

Known mechanisms for resistance to crizotinib include the gatekeeper L1196M mutation [6], other secondary *ALK* gene mutations (F1174L, C1156Y, G1202R, S1206Y, 1151-T-ins, and G1269A) [7, 8, 9, 10], *ALK* amplification [7], and activation of bypass signals via activation of other receptors (*KIT* amplification and epidermal growth factor receptor (EGFR) autophosphorylation) [8]. We recently reported that receptor ligands, such as epidermal growth factor (EGF), heparin binding-epidermal growth factor (HB-EGF), and transforming growth factor-α (TGF-α), also activate EGFR as a bypass signal and induce crizotinib resistance in EML4-ALK NSCLC cells [11].

Alectinib is a highly selective, new generation ALK-TKI that also has inhibitory activity against EML4-ALK NSCLC cells with the gatekeeper L1196M mutation [12]. In a clinical trial for crizotinib-treatment naïve NSCLC patients with *ALK* rearrangement, there was a response rate of 93.5% to alectinib [13]. Moreover, alectinib demonstrated promising effects, even in the crizotinib-treated NSCLC patients with *ALK* rearrangement [14]. While it is clear that resistance may also develop against this class of inhibitor, the mechanisms of resistance to alectinib are largely unknown.

Heat shock protein 90 (Hsp90) is a molecular chaperone that plays a central role in regulating the correct folding, stability, and function of numerous “client proteins,” including human epidermal growth factor receptor 2 (HER2), BRAF, mutant EGFR, and EML4-ALK, Bcr-Abl, Raf-1, which are required for cancer cell survival [15, 16, 17, 18]. Hsp90 inhibition is therefore thought to be a promising strategy for controlling tumors, including those of EML4-ALK NSCLC. A natural product, geldanamycin, was found to directly bind to the ATP-binding pocket in the N-terminal domain of Hsp90 and block the binding of nucleotides to Hsp90; hence, geldanamycin was found to inhibit Hsp90 function. The first water-soluble, semi-synthetic derivative of geldanamycin is 17-dimethylaminoethylamino-17-demethoxygeldanamycin (17-DMAG), which has shown excellent bioavailability and is quantitatively metabolized much less than other geldanamycin derivatives, such as 17-Allylamino 17-demethoxygeldanamycin (17-AAG) [19, 20].

In the present study, we examined whether receptor ligands would trigger resistance to a highly selective ALK-TKI, alectinib. Additionally, since we previously demonstrated that the Hsp90 inhibitor overcame EGFR-TKI resistance triggered by HGF, a ligand of MET, in *EGFR*-mutant lung cancer cells [21], we determined whether Hsp90 inhibition by 17-DMAG would overcome ligand-triggered alectinib resistance in *ALK*-rearranged NSCLC cells.

## RESULTS

### Exogenously added HGF and EGFR ligands induce resistance to alectinib in EML4-ALK NSCLC cells

**Figure 1:**
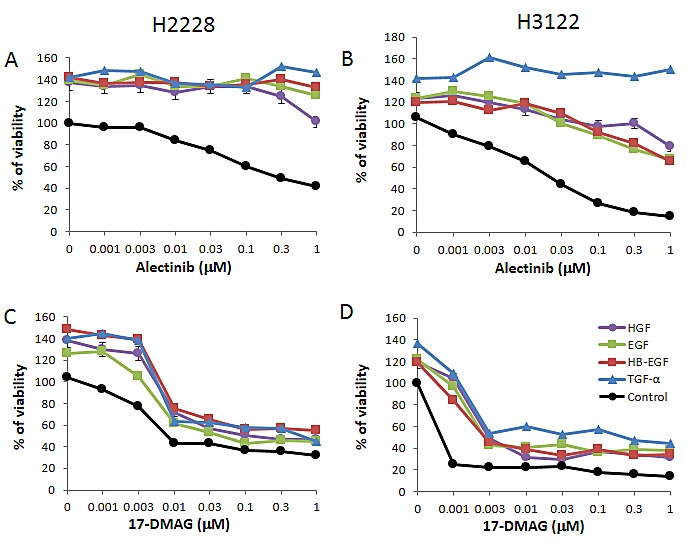
17-DMAG suppresses the growth of EML4-ALK NSCLC cells in the presence of HGF and EGFR ligands The EML4-ALK lung cancer cell lines human H2228 and human H3122 were treated with increasing concentrations of alectinib or 17-DMAG, with or without HGF (50 ng/mL), EGF (100 ng/mL), HB-EGF (10 ng/mL), and TGF-α (100 ng/mL), and cell viability was determined after 72 h by MTT assay. Data shown are representative of at least 3 independent experiments. Error bars indicate standard deviation (SD) of triplicate cultures.

Two EML4-ALK NSCLC cell lines, H2228 and H3122, were sensitive to crizotinib (IC50 0.3 μmol/L and 0.06 μmol/L, respectively). These cell lines were also sensitive to alectinib (IC50 0.24 μmol/L and 0.03 μmol/L, respectively). Exogenously added HGF and EGFR ligands (EGF, HB-EGF, and TGF-α) slightly stimulated cell growth, as determined by cell counting (Supplementary Fig. 1), and increased cell viability was determined by MTT assay (Fig. 1A-B). Under these experimental conditions, HGF and EGFR ligands remarkably reduced susceptibility of H2228 and H3122 cells to alectinib.

### 17-DMAG inhibits the viability of EML4-ALK NSCLC cells, irrespective of the presence of exogenously added HGF or EGFR ligands

Only the Hsp90 inhibitor 17-DMAG inhibited the viability of H2228 (Fig. 1C) and H3122 (Fig. 1D) cells in a dose-dependent manner. Importantly, 17-DMAG inhibited the viability of H2228 and H3122 cells, even in the presence of HGF or EGFR ligands. These results suggest that 17-DMAG may overcome alectinib resistance triggered by HGF or EGFR ligands, such as EGF, HB-EGF, and TGF-α.

### 17-DMAG inhibits the viability of EML4-ALK NSCLC cells in the presence of endogenous HGF

Recently, HGF was reported to induce resistance to various molecular-targeted drugs in various types of cancers with oncogene drivers [22, 23]. Moreover, our previous study reported that HGF was overexpressed in the *EGFR* mutant cancer cells that acquired resistance to EGFR-TKIs, indicating endogenous HGF production by cancer cells [24]. These findings suggest that HGF can be overexpressed in EML4-ALK NSCLC cells that acquire resistance to ALK inhibitors.

**Figure 2:**
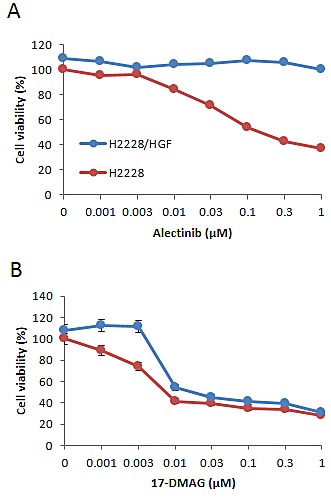
HGF-gene transfection resulted in reducing susceptibility of EML4-ALK NSCLC cells to alectinib but not 17-DMAG H2228/Vec (A) or H2228/HGF (B) cells were treated with increasing concentrations of alectinib or 17-DMAG, and cell viability was determined after 72 h by MTT assay. Data shown are representative of at least 3 independent experiments. Error bars indicate SD of triplicate cultures.

Therefore, we next examined whether endogenously expressed HGF induced alectinib resistance in EML4-ALK NSCLC cells. To assess this question, we generated stable HGF-gene transfectants in H2228 cells (H2228/HGF); as a control, we generated H2228/Vec cells transfected with vector alone. H2228/HGF cells secreted high concentrations of HGF (16.0 ± 0.4 ng/mL), whereas the HGF concentrations secreted by H2228 and H2228/Vec cells were under the detection limit. Consistent with the results of exogenously added HGF, HGF-transfected H2228 (H2228/HGF) cells became insensitive to alectinib (Fig. 2A), indicating that endogenously-expressed HGF also induced resistance to alectinib in EML4-ALK NSCLC cells.

We further found that 17-DMAG inhibited the growth of both H2228/Vec and H2228/HGF cells, because each had an IC50 of 0.01 μmol/L (Fig. 2B). These findings indicate that 17-DMAG may overcome alectinib resistance triggered by endogenously-produced HGF.

### HGF reduces alectinib susceptibility via MET phosphorylation, and 17-DMAG reduces expression of ALK and MET

**Figure 3:**
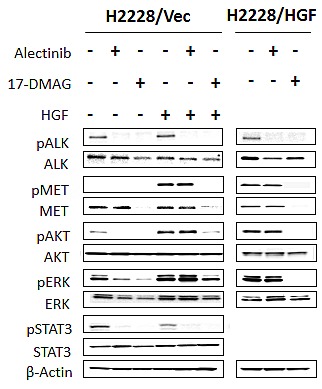
17-DMAG reduced MET protein expression and inhibited downstream pathways, even in the presence of HGF H2228/Vec or H2228/HGF cells were treated with or without alectinib (0.3 μmol/L) for 2 h or 17-DMAG (0.3 μmol/L) for 24 h and then stimulated with or without HGF (50 ng/mL) for 10 minutes. The resultant cells were lysed, and the indicated proteins were detected by immunoblotting. Data shown are representative of at least 3 independent experiments.

To explore the molecular mechanism by which HGF reduced susceptibility to alectinib and 17-DMAG inhibited cell growth, even in the presence of HGF, we examined the protein expression and phosphorylation status of MET, ALK, and their downstream molecules (PI3K/AKT, ERK1/2, and STAT3) by Western blotting (Fig. 3).Since HGF reduced alectinib susceptibility more potently in H2228 compared with H3122 cells (Fig. 1A-B), we mainly used H2228 cells in the following experiments. H2228 (data not shown) and H2228/Vec (Fig. 3) cells expressed ALK and MET proteins (ALK were phosphorylated but MET were not), as well as the downstream molecules AKT, ERK1/2, and STAT3. In the absence of HGF, alectinib inhibited ALK phosphorylation, thereby inhibiting AKT, ERK1/2, and STAT3 phosphorylation.

In the presence of HGF, alectinib failed to inhibit MET, AKT, and ERK1/2 phosphorylation, although it inhibited ALK and STAT3 phosphorylation. These results suggest that HGF reduced susceptibility to alectinib by mainly restoring AKT and ERK1/2 pathways via MET activation.

In parallel experiments, 17-DMAG decreased the expression of ALK and MET proteins and inhibited their phosphorylation and AKT, ERK1/2, and STAT3 phosphorylation, irrespective of HGF presence. Similar results were observed in H2228/HGF (Fig. 3) and H3122 cells (Supplementary Fig. 2). These results indicate that 17-DMAG decreases protein expression of ALK and MET, thereby suppressing downstream signaling and overcoming alectinib resistance caused by HGF.

### 17-DMAG reduces EGFR and AKT protein expression and inhibits downstream pathways, even in the presence of EGFR ligands

**Figure 4:**
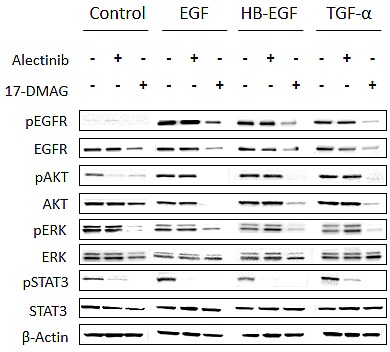
17-DMAG reduced MET protein expression and inhibited downstream pathways, even in the presence of EGFR ligands H2228 cells were treated with or without alectinib (0.3 μmol/L) for 2 h or 17-DMAG (0.3 μmol/L) for 24 h, and then stimulated with or without EGF (100 ng/mL), HB-EGF (10 ng/mL), and TGF-α (100 ng/mL) for 10 min. The resultant cells were lysed, and the indicated proteins were detected by immunoblotting. Data shown are representative of at least 3 independent experiments.

We also examined the protein expression and phosphorylation status of EGFR and its downstream molecules in H2228 cells stimulated with EGFR ligands(Fig. 4). H2228 expressed EGFR, but EGFR was not constitutively phosphorylated in our experimental conditions. The EGFR ligands EGF, HB-EGF, and TGF-α remarkably induced EGFR phosphorylation. In these experimental conditions, alectinib failed to inhibit phosphorylation of EGFR or downstream AKT and ERK1/2, while it inhibited STAT3 phosphorylation. These results suggest that EGFR ligands reduced susceptibility to alectinib mainly by restoring AKT and ERK1/2 pathways via EGFR activation.

On the other hand, 17-DMAG decreased EGFR protein expression, resulting in inhibition of AKT, ERK1/2, and STAT3 phosphorylation, irrespective of the presence of EGFR ligands. These results suggest that 17-DMAG decreases EGFR protein expression, thereby suppressing downstream signaling and overcoming alectinib resistance triggered by EGFR ligands.

### 17-DMAG induces apoptosis of EML4-ALK lung cancer cells, even in the presence of HGF

**Figure 5:**
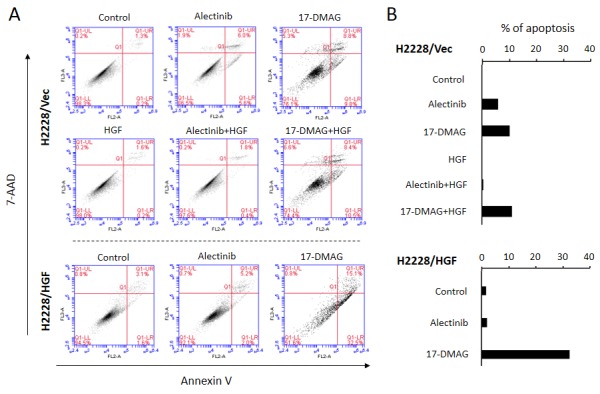
17-DMAG induced apoptosis of EML4-ALK NSCLC cells, even in the presence of HGF A. Apoptotic cells were evaluated by the 7-AAD cell viability assay, as described in the Materials and Methods. B. Quantification of apoptotic cells.

We next assessed whether alectinib and 17-DMAG induced H2228/Vec cell apoptosis in the absence or presence of HGF. Alectinib induced apoptosis of H2228/Vec cells in the absence, but not presence, of HGF (Fig. 5). In contrast, 17-DMAG induced apoptosis in both the presence and absence of HGF. In a similar fashion, 17-DMAG, but not alectinib, induced H2228/HGF cell apoptosis.

### 17-DMAG inhibits H2228 cell viability, even in the presence of both of HGF and EGFR ligands

**Figure 6:**
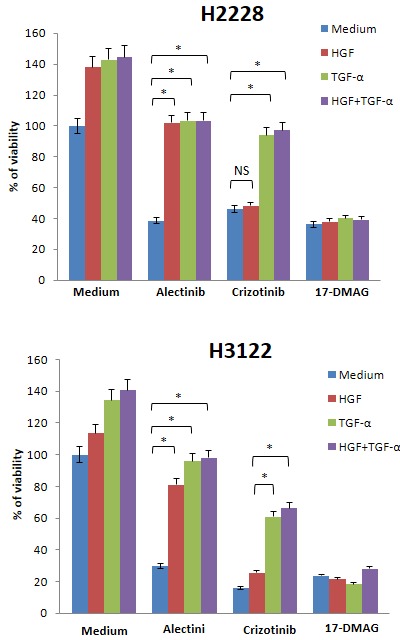
17-DMAG reduced viability of EML4-ALK NSCLC cells, even in the presence of both HGF and TGF-α H2228 and H3122 cells were incubated with or without alectinib (0.1 μmol/L), crizotinib (0.1 μmol/L), and/or HGF (50 ng/mL) and TGF-α (100 ng/mL), and cell viability was determined after 72 h by MTT assay. The percentage of cell viability is shown relative to controls without HGF or TGF-α treatment. *, *P* < 0.001 (one-way ANOVA). NS, not significant. Data shown are representative of at least 3 independent experiments. Error bars indicate SD of triplicate cultures.

Since several growth factors can be simultaneously produced in cancer microenvironments [25, 26], it is possible that HGF and EGFR ligands are co-expressed in EML4-ALK NSCLC cells. Crizotinib inhibits MET, ALK, and ROS1, and it is supposed to overcome alectinib resistance caused by HGF alone. We therefore examined the effect of 17-DMAG compared with crizotinib in the presence of HGF plus EGFR ligands. H2228 and H3122 cells became insensitive to alectinib in the presence of HGF, TGF-α, and HGF with TGF-α (Fig. 6). These cells were sensitive to crizotinib in the presence of HGF, but they became much less sensitive to crizotinib in the presence of TGF-α with or without HGF. However, H2228 and H3122 were sensitive to 17-DMAG in the presence of HGF, TGF-α, or HGF with TGF-α. These results suggest that 17-DMAG may overcome alectinib resistance, even in the presence of ligands for two different receptors.

## DISCUSSION

We demonstrated that ligands of MET (HGF) and EGFR (EGF, HB-EGF, and TGF-α) triggered resistance to alectinib in *ALK*-rearranged NSCLC cells, and that the Hsp90 inhibitor 17-DMAG overcame the resistance triggered by these receptor ligands. 17-DMAG inhibited protein levels of ALK, EGFR, and MET, even in the presence of ligand activation, and suppressed of AKT and ERK1/2 phosphorylation, thereby inducing apoptosis of *ALK*-rearranged NSCLC cells, irrespective of the presence of HGF or EGFR ligands. Since the Hsp90 inhibitor by itself could inhibit both driver (from rearranged *ALK*) and resistance signals (from activated receptors; MET and EGFR), it may be an ideal agent for overcoming ligand-triggered alectinib resistance in *ALK*-rearranged NSCLC.

Activation of bypass signals is a common resistance mechanism for targeted drugs. For example, EGFR-TKI resistance could be caused by *MET* amplification [27], HGF-triggered MET activation [23], Gas6-triggered AXL activation [28], and *HER2* amplification [29] in *EGFR* mutant lung cancer. BRAF inhibitor resistance could be caused by HGF-triggered MET activation [30], and IGF-1 triggered its receptor activation [31] in *BRAF* mutant melanoma. Crizotinib resistance could be caused by EGFR ligand-triggered EGFR activation [11], and stem cell factor (SCF)-triggered amplified cKIT activation [8] in EML4-ALK NSCLC. Therefore, HGF and EGFR ligands may be common resistance triggers that activate bypass survival signal via their receptor activation. The results in the present study are consistent with previous research indicating that alectinib resistance was induced by HGF and EGFR ligands.

Previous studies reported that several signaling pathways, including PI3K/AKT, MEK/ERK, and STAT3, are essential for survival and/or resistance to ALK inhibitors in *ALK*-rearranged NSCLC cells [12, 32]. Accordingly, we found that alectinib inhibited STAT3 and ALK phosphorylation. In the presence of alectinib, HGF or EGFR ligands restored AKT and ERK1/2, but not STAT3, phosphorylation and thereby made EML4-ALK cells insensitive to alectinib. These observations indicate that, when activated by their ligands, AKT and ERK signals from MET or EGFR play pivotal roles in alectinib resistance of EML4-ALK NSCLC cells.

It is of interest in the present study that HGF and EGFR ligands induced not only ALK-TKI resistance but also increased cell growth of EML4-ALK NSCLC cells. HGF and EGFR ligands also induced morphological change of H2228 cells (Supplementary Fig. 3). Therefore, these receptor ligands may modulate various cancer phenotypes of EML4-ALK NSCLC cells. HGF-MET and EGFR-ligands-EGFR axises play pivotal roles in progression of various types of tumors (33, 34). We are planning further studies to explore the molecular mechanisms of this morphological change and co-relation between the expression of receptor ligands in patient specimens and clinical characteristics in *ALK*-rearranged NSCLC.

Inter- and/or intra-tumor heterogeneity is a critical obstacle in cancer therapy with targeted drugs [35]. This is also the case in ALK-TKI resistance. Intra-tumor heterogeneity caused by crizotinib resistance results from the L1196M gatekeeper *ALK* mutation, and other *ALK* secondary C1156Y mutations co-existed in malignant pleural effusion of a patient who acquired crizotinib resistance [6]. Moreover, activation of different two receptors, EGFR and amplified *KIT* (both of which could induce crizotinib resistance), also co-existed in one crizotinib-resistant tumor [8].

Hsp90 inhibitors have been reported to overcome crizotinib resistance caused by several mechanisms, including *ALK* amplification, L1196M gatekeeper *ALK* mutation, other secondary *ALK* mutations (including F1174L), and epithelial to mesenchymal transition. Furthermore, we demonstrated that the Hsp90 inhibitor may overcome alectinib resistance, even when ligands of MET and EGFR co-exist. A new generation of Hsp90 inhibitors, including ganetespib, has recently been developed, and remarkable efficacy has been reported in a co-clinical model and early phase clinical studies [36]. Therefore, Hsp90 inhibition using new generation inhibitors may be a promising strategy to treat *ALK*-rearranged NSCLCs that acquire resistance to alectinib.

## MATERIALS AND METHODS

### Cell culture

The H2228 human lung adenocarcinoma cell line, with EML4-ALK fusion protein variant3 (E6;A20), was purchased from the American Type Culture Collection (Manassas, VA). The H3122 human lung adenocarcinoma cell line, with EML4-ALK fusion protein variant1 (E13;A20), was kindly provided by Dr. Jeffrey A. Engelman of the Massachusetts General Hospital Cancer Center (Boston, MA) [37]. H2228 and H3122 cells were cultured in RPMI-1640 medium supplemented with 5% fetal bovine serum (FBS), penicillin (100 U/mL), and streptomycin (50 μg/mL) in a humidified CO_2_ incubator at 37°C. All cells were passaged for less than 3 months before renewal from frozen, early-passage stocks obtained from the indicated sources. Cells were regularly screened for *Mycoplasma* using a MycoAlert *Mycoplasma* Detection Kit (Lonza, Basel, Swiss).

### Reagents

Alectinib, crizotinib, and 17-DMAG were purchased from Seleck Chemicals(Houston, TX). Recombinant EGF, TGF-α, and HB-EGF were purchased from R&D Systems(Minneapolis, MN). Recombinant HGF was prepared as described in a previous study [38].

### Cell growth assay

Cell proliferation was measured using the 3-(4,5-dimethylthiazol-2-yl)-2,5 diphenyl terazolium bromide (MTT) dye reduction method [39]. Tumor cells were harvested at 80% confluence, seeded at 2 × 10^3^ cells per well in 96-well plates, and incubated in appropriate medium for 24 h. Several concentrations of alectinib, crizotinib, 17-DMAG, and/or EGF, TGF-α, HB-EGF, and HGF were added to each well, and incubation continued for another 72 h. Fifty μL MTT (2 mg/mL; Sigma, St.Louis, MO) was added to each well, followed by incubation for 2 h at 37°C. The media were removed and the dark blue crystals in each well were dissolved in 100 μL of dimethyl sulfoxide (DMSO). Absorbance was measured with an MTP-120 Microplate reader (Corona Electric, Hitachinaka, Ibaraki, Japan) at test and reference wavelengths of 550 and 630 nm, respectively. The percentage growth was calculated relative to untreated controls. Each assay was carried out at least in triplicate, with results based on 3 independent experiments.

### HGF-gene transfection

One day before transfection, aliquots of 1 × 10^5^ H2228 cells in 1 mL of antibiotic-free medium were plated on 6-well plates. The full-length *HGF* cDNA cloned into the BCMGSneo expression vector [40] was transfected using Lipofectamine 2000 according to the manufacturer’s instructions. After incubation for 24 h, the cells were washed with phosphate buffered saline (PBS)and incubated for an additional 72 h in antibiotic-containing medium. Then, the cells were selected in G418 sulfate (Calbiochem, Jolla, CA). After limiting dilution, the HGF-producing cells, H2228/HGF, were established. HGF production by H2228/HGF was confirmed by enzyme linked immunosolvent assay (ELISA).

### HGF production

Cells (2 × 10^5^) were cultured in RPMI-1640 medium with 10% FBS for 24 h. The cells were washed with PBS and incubated for 48 h in 2 mL of RPMI-1640 medium with 10% FBS. Then, culture medium was harvested and centrifuged, and the supernatant was stored at −70°C until analysis. HGF concentrations were determined by IMMUNIS HGF EIA (Institute of Immunology, Tokyo) according to the manufacturer’s protocols. All samples were run in duplicate. Color intensity was measured at 450 nm using a spectrophotometric plate reader. Growth factor concentrations were determined by comparison with standard curves, and the HGF detection limit was 100 pg/mL.

### Apoptosis assay

Cell apoptosis induced by alectinib and 17-DMAG was measured by the PE Annexin V Apoptosis Detection Kit I (BD Biosciences, San Jose, CA) which detects and quantifies apoptotic cells with phycoerythrin (PE) Annexin V and 7-amino-actinomycin (7-AAD) staining. Cells were analyzed on a FACSCalibur flow cytometer with CellQuest software (Becton Dickinson, Franklin Lakes, NJ).

### Western blotting

Sodium dodesyl sulfate (SDS) polyacrylamide gels (Bio-Rad, Hercules, CA) were loaded with 40 μg total protein per lane; following electrophoresis, the proteins were transferred onto polyvinylidene difluoride membranes (Bio-Rad), which were incubated with Blocking One (Nacalai Tesque, Kyoto, Japan) for 1 h at room temperature, followed by overnight incubation at 4°C with anti-ALK (C26G7), anti-phospho-ALK (Tyr1604), anti-phospho-EGFR (Tyr1068), anti-STAT3(79D7), anti-phospho-STAT3 (Y705), anti-AKT, anti-phospho-AKT (Ser473), anti-ErbB4 (111B2), anti-phospho-ErbB4 (Tyr1284), anti-MET (25H2), anti-phospho-MET (Y1234/Y1235) (3D7), or anti-β-actin (13E5) antibodies (1:1,000 dilution each; Cell Signaling Technology, Danvers, MA), or with anti-human EGFR (1 μg/mL), anti-human/mouse/rat extracellular signal-regulated kinase (Erk)1/Erk2 (0.2 μg/mL), or anti-phospho-Erk1/Erk2 (T202/Y204) (0.1 μg/mL) antibodies (R&D Systems). After washing 3 times, the membranes were incubated for 1 h at room temperature with secondary antibodies (horseradish peroxidase-conjugated species-specific antibodies).

Immunoreactive bands were visualized with SuperSignal West Dura Extended Duration Substrate Enhanced Chemiluminescent Substrate (Pierce, Osaka, Japan). Each experiment was independently carried out at least 3 times.

### Statistical analysis

Differences were analyzed by one-way ANOVA. All statistical analyses were carried out using GraphPad StatMate 4 (GraphPad Software, Inc., San Diego, CA). *P* < 0.05 was considered significant.

## SUPPLEMENTARY FIGURES


