# Supplementary material for: Receptor ligand-triggered resistance to alectinib and its circumvention by Hsp90 inhibition in EML4-ALK lung cancer cells
Source: Oncotarget. 2014 Jun 3;5(13):4920–8. doi: 10.18632/oncotarget.2055 (PMC4148110; doi:10.18632/oncotarget.2055)
Supplement: Supplementary file 1 [file oncotarget-05-4920-s001.pdf]

Receptor ligand-triggered resistance to alectinib and its circumvention by Hsp90 inhibition in EML4-ALK lung cancer cells

Supplementary Material

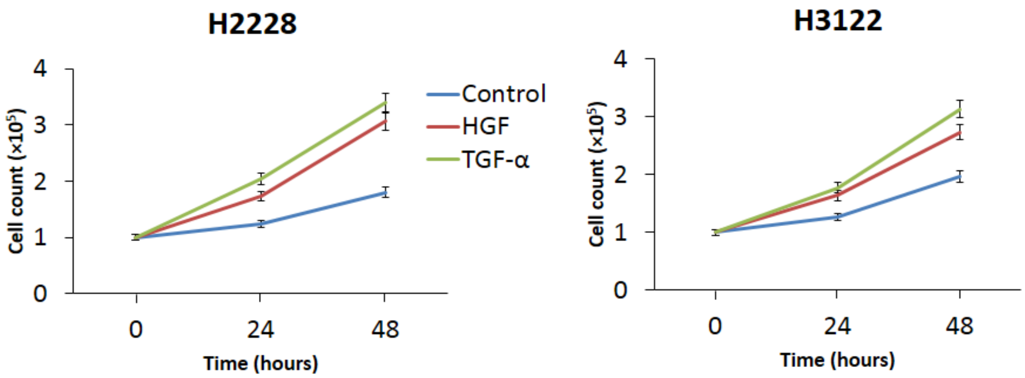

Supplementary Figure 1: HGF and EGFR ligands slightly stimulated cell growth of H2228 and H3122 cells. H2228 and H3122 cells were incubated with or without HGF (50 ng/mL) or TGF-α (100 ng/mL). Then, the cells were harvested, and cell number was counted after trypan blue staining.

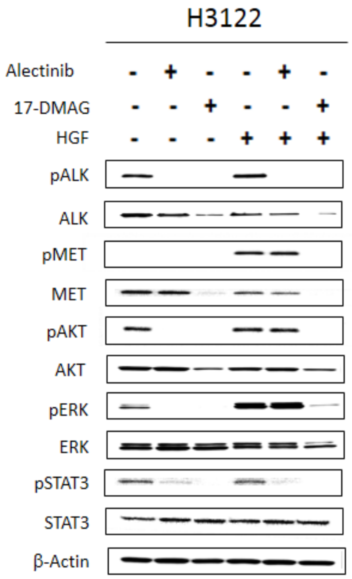

Supplementary Figure 2: 17-DMAG reduced MET protein expression and inhibited downstream pathways, even in the presence of EGFR ligands in H3122. H3122 cells were treated with or without alectinib (0.3 μmol/L) for 2 h or 17-DMAG (0.3 μmol/L) for 24 h, and then stimulated with or without HGF (50 ng/mL) for 10 minutes. The resultant cells were lysed, and the indicated proteins were detected by immunoblotting. Data shown are representative of at least 3 independent experiments.

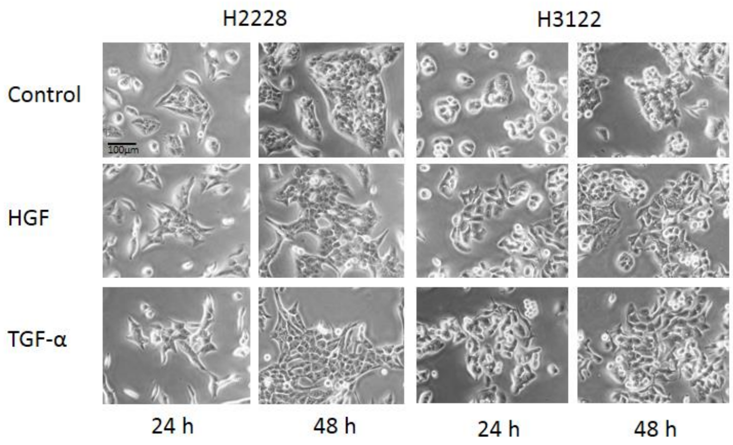

Supplementary Figure 3: Light micrographs of cellular morphology of H2228 and H3122 cells treated with HGF or TGF-α. H2228 and H3122 cells were incubated with HGF (50 ng/mL) or TGF-α (100 ng/mL) for 24 or 48 h.
